# The knowledge of and educational interest in sexual medicine among Finnish medical and midwifery students: A web-based study

**DOI:** 10.18332/ejm/186401

**Published:** 2024-05-15

**Authors:** Sanna-Mari Manninen, Päivi Polo-Kantola, Markus Riskumäki, Tero Vahlberg, Katja Kero

**Affiliations:** 1 Department of Obstetrics and Gynecology, Turku University Hospital and University of Turku, Turku, Finland; 2 Department of Health Promotion, Metropolia University of Applied Sciences, Helsinki, Finland; 3 Unit of Biostatistics, Department of Clinical Medicine, Faculty of Medicine, University of Turku, Turku, Finland

**Keywords:** knowledge, medical student, midwifery student, sex education, sexual medicine, questionnaire

## Abstract

**INTRODUCTION:**

Many elements of life can affect sexual health; thus, healthcare professionals require good knowledge of sexual medicine to encounter patients with these issues. We aimed to study final-year medical and midwifery students’ self-reported knowledge of factors associated with sexuality and their knowledge of how to evaluate and treat/counsel patients with sexual problems. In addition, educational interests regarding sexual medicine were assessed.

**METHODS:**

In a cross-sectional study, a web-based questionnaire was distributed to final-year medical (n=233) and midwifery (n=131) students graduating between December 2018 and May 2019 in Finland.

**RESULTS:**

Both student groups self-reported insufficient knowledge of how to consider sexuality in mentally ill patients, how to encounter victims of domestic violence/sexual abuse, and how multiculturalism affects sexuality. In addition, compared to the midwifery students, the medical students were more likely to self-report insufficient knowledge of the basics of sexual pleasure and treating the lack of it (p<0.001), including how to treat sexual problems due to relationship problems (p<0.001) or chronic diseases (p=0.015). Although several educational areas of interest arose, both student groups had two mutual most desirable educational interests: 1) reasons for dyspareunia and its treatment, n=117/233 (50.2%) for medical students, and n=60/131 (45.8%) for midwifery students; and 2) lack of sexual desire and its treatment, n=100/233 (42.9%) for medical students, and n=55/131 (42.0%) for midwifery students.

**CONCLUSIONS:**

In both student groups, the self-reported knowledge of sexual medicine was insufficient. Thus, more education on sexual medicine should be included in the curricula of medical and midwifery education.

## INTRODUCTION

Sexual rights are human rights, which include the right to life, the right to be free from torture, the right to health, the right to privacy, freedom from discrimination, and, importantly, the right to sexual education and information^1^. Because sexuality is an essential part of being human, healthcare professionals must be knowledgeable about it. Many elements of life can affect sexual health, such as aging^2,3^, poor physical health^2,4^, childbirth^5^ and dissatisfaction with one’s sex life^2,6^. In addition, several chronic diseases may directly or through treatment medications have a notable negative effect^4,7-9^. Accordingly, physicians, midwives, and other healthcare professionals should have good knowledge of sexual medicine. Sufficient education in medical and midwifery schools is therefore necessary.

Although issues concerning sexual and reproductive health are currently taught in various subjects in medical schools, more detailed sexual issues are often overlooked in the curricula^10^. In midwifery schools, more sexual and reproductive health studies are included in their curricula; however, overviews of important sexual issues are missing^11^. Contraceptive appointments, for instance, are convenient for sexual guidance, but final-year midwifery students have reported receiving too little education on this issue and having difficulties utilizing theoretical knowledge in practice^12^. Midwives are particularly likely to meet women with sexual issues; hence, these issues should be included widely in the curriculum – for example, instructing women on the use of local estrogen for vulvovaginal dryness experienced during postpartum and climacteric, and advising couples on how to practice intimacy if pregnancy has some complications. In addition, midwives have an important task in sexual and reproductive health counseling and education not only for women but also within the family and community^13^. Nonetheless, in previous studies, a lack of knowledge and education on sexual medicine has been reported regarding medical^14-17^ and midwifery^11,12,18-21^ education.

In this Sexual Medicine Education (SexMEdu) sub-study, we studied final-year medical and midwifery students’ self-reported knowledge of: 1) factors associated with sexuality, and 2) evaluations and treatment of patients with sexual problems. In addition, we aimed to study the students’ educational interest in sexual medicine. In medical school, the education concerning sexual issues is typically called ‘sexual medicine’, and in midwifery school, the term is ‘sexual and reproductive health’. For consistency, the term ‘sexual medicine’ is used throughout this article for medical and midwifery education.

## METHODS

### Subjects

This was a cross-sectional study. The participants were medical and midwifery students graduating between December 2018 and May 2019 in Finland. All five faculties of medicine educating medical students and all eight universities of applied sciences educating midwifery students in Finland took part in the study. We chose to compare medical students to midwifery students, as we hypothesized that the education in midwifery programs is directed more towards sexual health issues than in other nursing programs. During the study period, 562 medical students graduated, of whom 233 participated in the study, giving a response rate of 41.5%. Concerning the midwifery students, 253 graduated, of whom 131 participated, giving a response rate of 51.8%. The mean age of the medical students was 28.5 years (SD= 4.0, range: 24–48 years), and 60.5% (n=141) were women and 39.5% (n=92) were men. Of the midwifery students, all (n=131) were women, and their mean age was 28.1 years (SD=5.0, range 23–50 years).

### Ethics

Replying to the questionnaire implied consent, which was explained in the introduction of the questionnaire. The study protocol was approved by the Ethics Committee of Turku University (44/2017).

### Questionnaires

The questionnaire included 17 items, which we adopted and slightly modified from the Portuguese SEXOS study^22^ of general practitioners and a German study^23^ of medical students. Permissions to use the questionnaires were obtained from the researchers. We also used the Portuguese questionnaire in our earlier SexMEdu sub-studies of general practitioners^24,25^, and the Portuguese and German questionnaires were used in a sub-study of medical and midwifery students^26^. We piloted the questionnaire with 27 medical students and used their feedback to amend the content. This part of the SexMEdu sub-study consisted of three independent fields.

*Field A: Self-reported knowledge of factors associated with sexuality*

This field involved the following 8 items: 1) I know the basics of human sexual development, 2) I know the basics of sexual evolution and expression of sexuality across the lifespan, 3) I know the basics of aging and sexuality, 4) I know how to consider sexuality when taking care of patients with mental illnesses, 5) I know the basics of how multiculturalism affects sexuality, 6) I know how to encounter victims of domestic violence/sexual abuse, 7) My knowledge of sexual orientation (e.g. hetero/homosexuality) is adequate, and 8) My knowledge of gender diversity (e.g. transgender people) is adequate. The items were rated on a 5-point scale (1=totally agree to 4=totally disagree, with 5=cannot say).

*Field B: Self-reported knowledge of evaluating and treating patients with sexual problems*

This field involved the following 8 items: 1) I know the basics of sexual pleasure and treating lack of it, 2) I know the basics of decreased libido and the basics of its treatment, 3) I know the diagnostics and treatment of arousal problems, 4) I know the diagnostics and treatment of orgasm disorders, 5) I know the diagnostics and treatment of dyspareunia, 6) I know the basics of erectile dysfunction and the basics of its treatment, 7) I know how to treat sexual problems due to relationship problems, and 8) I know how to treat sexual problems due to chronic diseases. The items were rated on a 5-point scale (1=totally agree to 4=totally disagree, with 5=cannot say).

*Field C: Educational interests in sexual medicine*

This field involved the following 25 items for which the students needed to choose 1–5 that they considered the most interesting areas requiring more education: 1) sexual development during childhood and adolescence, 2) sexual history taking, 3) hormones affecting sexuality, 4) arousal problems, 5) reasons for and treatment of erectile dysfunction, 6) reasons for and treatment of premature ejaculation, 7) reasons for and treatment of priapism, 8) sexual orientation, 9) development of gender and gender identity, 10) surgery of sex organs, 11) reasons for and treatment of orgasm disorders, 12) reasons for and treatment of dyspareunia, 13) medications affecting sexuality, 14) lack of sexual desire and its treatments, 15) sexual abuse of a child, 16) sexual violence/sexual abuse of an adult, 17) effects of infertility and its treatment on sexuality, 18) effects of abortion on sexuality, 19) effects of relationship problems on sexuality, 20) sociological and cultural factors affecting sexuality, 21) different methods of sexual interaction/sexual habits, 22) disability and sexuality, 23) aging and sexuality, 24) social media and sexuality, and 25) something else. If more options than five were marked, the program asked the participant to reduce the amount in order to return the questionnaire.

### Statistical analysis

Data are described using frequencies (percentages). In the analyses, each item in fields A and B was dichotomized (‘totally agree or agree’ vs ‘disagree or totally disagree’), and the ‘cannot say’ responses were omitted from the analyses. In all comparisons, the medical students were compared to the midwifery students. The associations between the students’ age and the A and B fields of interest were analyzed using multivariable logistic regression (examining each item separately in each field in the analyses). In addition, the gender associations were carried out in the age-adjusted sub-analysis of the medical students, as all the midwifery students were women. The differences between medical and midwifery students are presented using age-adjusted odds ratios (AORs) with 95% confidence intervals (CIs). A p<0.05 was considered statistically significant, and statistical analyses were performed using the SAS System for Windows, version 9.4 (SAS Institute Inc., Cary, NC).

## RESULTS

*Field A: Self-reported knowledge of factors associated with sexuality*

The data of the self-reported knowledge of factors associated with sexuality are described in Table 1. All presented data were adjusted by age. The medical and midwifery students reported insufficient knowledge of how to consider sexuality when caring for patients with mental illnesses, how to encounter victims of domestic violence/sexual abuse, and how multiculturalism affects sexuality; in all these, more than 50% of students in both groups reported having insufficient knowledge. However, compared to the midwifery students, the medical students were more likely to report insufficient knowledge of the basics of human sexual development (AOR=4.90; 95% CI: 2.54–9.46, p<0.001), the basics of sexual evolution and expressions of sexuality across the lifespan (AOR=4.52; 95% CI: 2.57–7.94, p<0.001), the basics of aging and sexuality (AOR=5.03; 95% CI: 2.80–9.04, p<0.001), the basics of how multiculturalism affects sexuality (AOR=5.09; 95% CI: 3.06–8.47, p<0.001), and gender diversity, e.g. transgender people (AOR=2.48; 95% CI: 1.54–4.01, p<0.001). Neither the students’ age nor the medical students’ gender showed an association (data not shown).

**Table 1:** The final-year medical and midwifery students’ self-reported knowledge of factors associated with sexuality. For the cross-sectional study, the students graduated between December 2018 and May 2019 in Finland

	*Medical students (N=233)*	*Midwifery students (N=131)*			
	*Disagree or totally disagree % (n/N)*	*Disagree or totally disagree % (n/N)*	*AOR*	*95% CI*	*p*
I know the basics of human sexual development	32.8 (75/229)	9.2 (12/131)	4.90	2.54–9.46	<0.001
I know the basics of sexual evolution and expression of sexuality across the lifespan	42.3 (96/227)	14.0 (18/129)	4.52	2.57–7.94	<0.001
I know the basics of aging and sexuality	41.2 (94/228)	12.2 (16/131)	5.03	2.80–9.04	<0.001
I know how to consider sexuality when taking care of patients with mental illnesses	69.3 (158/228)	62.9 (78/124)	1.34	0.84–2.12	0.216
I know the basics of how multiculturalism affects sexuality	85.6 (190/222)	53.9 (70/130)	5.09	3.06–8.47	<0.001
I know how to encounter victims of domestic violence/sexual abuse	57.9 (124/214)	56.3 (67/119)	1.08	0.69–1.70	0.740
My knowledge of sexual orientation (e.g. hetero/ homosexuality) is adequate	21.2 (48/226)	14.8 (19/128)	1.55	0.86–2.77	0.142
My knowledge of gender diversity (e.g. transgender people) is adequate	45.1 (102/226)	25.0 (32/128)	2.48	1.54–4.01	<0.001

In all comparisons, the medical students were compared to the midwifery students. AOR: adjusted odds ratio. AOR higher than 1 indicates higher insufficiency of knowledge (two categories: agree or totally agree versus disagree or totally disagree) for the medical students. AOR less than 1 indicates higher insufficiency of knowledge for the midwifery students. ORs were adjusted for age. Multivariable logistic regression (‘cannot say’ responses were omitted from analyses).

*Field B: Self-reported knowledge of evaluating and treating patients with sexual problems*

The medical students self-reported insufficient knowledge in all assessed areas except the basics of erectile dysfunction and its treatment. The self-reported knowledge among the midwifery students was also insufficient except for the basics of sexual pleasure and treating lack of it. Compared to the midwifery students, the medical students were more likely to report insufficient knowledge of the basics of sexual pleasure and treating lack of it (AOR=3.66; 95% CI: 2.32–5.76, p<0.001), how to treat sexual problems due to relationship problems (AOR=2.96; 95% CI: 1.82–4.81, p<0.001), and how to treat sexual problems due to chronic diseases (AOR=1.89; 95% CI: 1.13–3.17, p=0.015). In contrast, compared to the midwifery students, the medical students reported less often insufficient knowledge of the basics of erectile dysfunction and its treatment (AOR=0.14; 95% CI: 0.08–0.22, p<0.001) (Table 2). In the sub-analysis of the medical students, compared to the male students, the female students were more likely to report insufficient knowledge of the basics of erectile dysfunction and its treatment (AOR=1.97; 95% CI: 1.04–3.75, p=0.038) and how to treat sexual problems due to chronic diseases (AOR=2.24; 95% CI: 1.12–4.48, p=0.022), whereas the male students were more likely to report insufficient knowledge of how to treat sexual problems due to relationship problems (AOR=2.24; 95% CI: 1.06–4.73, p=0.035). The students’ age showed no association (data not shown).

**Table 2:** The final-year medical and midwifery students’ self-reported knowledge of evaluating and treating patients with sexual problems. For the cross-sectional study, the students graduated between December 2018 and May 2019 in Finland

	*Medical students (N=233)*	*Midwifery students (N=131)*			
	*Disagree or totally disagree % (n/N)*	*Disagree or totally disagree % (n/N)*	*AOR*	*95% CI*	*p*
I know the basics of sexual pleasure and treating lack of it	65.8 (148/225)	34.4 (45/131)	3.66	2.32–5.76	<0.001
I know the basics of decreased libido and the basics of its treatment	75.8 (175/231)	58.9 (76/129)	2.18	1.37–3.46	0.001
I know the diagnostics and treatment of arousal problems	93.4 (212/227)	92.9 (118/127)	1.08	0.46–2.56	0.859
I know the diagnostics and treatment of orgasm disorders	92.1 (210/228)	91.4 (117/128)	1.10	0.50–2.41	0.812
I know the diagnostics and treatment of dyspareunia	67.5 (156/231)	66.9 (87/130)	1.02	0.64–1.61	0.946
I know the basics of erectile dysfunction and the basics of its treatment	26.8 (61/228)	72.3 (94/130)	0.14	0.08–0.22	<0.001
I know how to treat sexual problems due to relationship problems	80.7 (180/223)	58.9 (76/129)	2.96	1.82–4.81	<0.001
I know how to treat sexual problems due to chronic diseases	82.2 (185/225)	71.1 (91/128)	1.89	1.13–3.17	0.015

In all comparisons, the medical students were compared to the midwifery students. AOR: adjusted odds ratio. AOR higher than 1 indicates higher insufficiency of knowledge (two categories: agree or totally agree versus disagree or totally disagree) for the medical students. AOR less than 1 indicates higher insufficiency of knowledge for the midwifery students. ORs were adjusted for age. Multivariable logistic regression (‘cannot say’ responses were omitted from analyses).

*Field C: Educational interests in sexual medicine*

**Figure 1:**
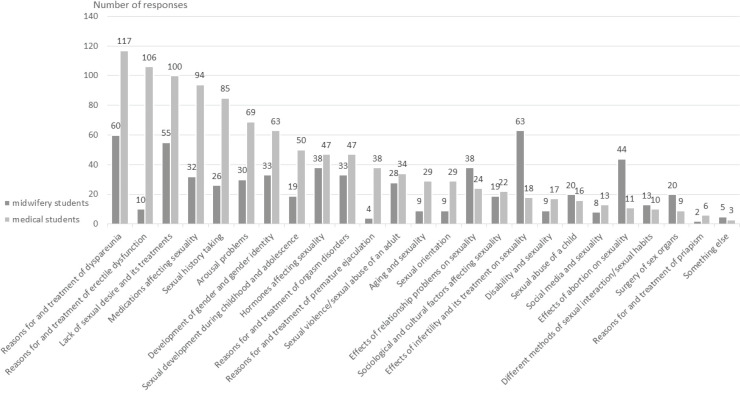
The final-year medical students’ (n=233) and midwifery students’ (n=131) responses to educational interests in sexual medicine (1–5 options could be chosen). For the cross-sectional study, the students graduated between December 2018 and May 2019 in Finland

The results of educational areas of interest are illustrated in Figure 1. All the areas drew at least some level of interest between both student groups. The top three areas of interest among the medical students (n=233) were: 1) the reasons for dyspareunia and its treatment (n=117; 50.2%), 2) the reasons for erectile dysfunction and its treatment (n=106; 45.5%); and 3) lack of sexual desire and its treatment (n=100; 42.9%). Among the midwifery students (n=131), the top three areas were: 1) the effects of infertility and its treatment on sexuality (n=63; 48.1%), 2) the reasons for dyspareunia and its treatment (n=60; 45.8%), and 3) the lack of sexual desire and its treatment (n=55; 42.0%).

## DISCUSSION

Our study aimed to compare the knowledge of sexual medicine between medical and midwifery students. We also explored the educational areas of the highest interest to the students concerning sexual medicine, the results of which can be utilized when planning the curricula for both student groups. We found that both medical and midwifery students self-reported insufficient knowledge of several factors associated with sexuality, most of whom were medical students. Concerning the evaluation and treatment of various sexual problems, self-reported knowledge insufficiency was even more profound in both groups and, again, more pronounced among the medical students. All the surveyed areas of sexual medicine aroused the participants’ attention, proposing a wide interest in the topics. Our results show the two different pathways that should be considered when developing education about sexual medicine in these two medical fields. The interests of the medical students mostly concerned subjects dealing with the reasons and treatments, likely because physicians focus primarily on diagnosing and treating various problems. The midwifery students were likewise interested in the reasons and treatments, but they were also interested in subjects dealing with counseling, such as infertility and abortion.

In our study, the most important areas of insufficient knowledge were: sexuality in patients with mental illness, encountering victims of domestic violence/sexual abuse, and understanding how multiculturalism affects sexuality. These results were found in both student groups. Mental health problems^27-29^, domestic violence and/or sexual abuse^27-29^, and gynecological symptoms^27,28^ are often found in the same patients, emphasizing the importance of considering sexual issues in these patients. Similar to our study, previous research on general practitioners^30^ showed a lack of knowledge concerning sexual abuse, likely hindering these issues from being addressed. In an Austrian study^14^ of 391 medical students, only 25% recalled having learned about sexual violence at least to some extent. Further, in a UK study^31^ of 372 midwives, most respondents had little, if any, education on the topic of sexual abuse and felt unable to deal effectively with it. Similarly, a text-based analysis of written material from a midwifery program in Sweden showed a lack of important subjects, including non-consensual sex (rape), honor-related violence, and the selling and buying of sex^11^.

Self-reported knowledge related to evaluating and treating/counseling patients with sexual problems was insufficient in almost every area evaluated in both student groups. Addressing patients’ sexuality requires, in addition to knowledge, good communication and practical skills. Importantly, physicians and midwives are key professionals in giving sexual and reproductive health counseling and education to their patients; however, in a qualitative Swedish study^20^, midwives reported a lack of knowledge and counseling tools as reasons for not addressing a patient’s sexuality. Implementing theoretical knowledge into practice is often challenging, and our findings highlight the insufficiency of sexual medicine education. The diagnostics and treatment of arousal problems and orgasm disorders were especially unclear among most participants. According to a systematic review and meta-analysis^32^, during the COVID-19 pandemic, there was a significant decrease in sexual function among sexually active adult women. The most affected areas of sexual function were arousal, orgasm, satisfaction, and pain during intercourse^32^. The self-reported knowledge of all these female sexual health aspects was assessed as insufficient by our participants, except for sexual pleasure, as reported by the midwifery students.

An important finding in our study was the self-reported insufficiency in knowledge regarding the treatment of sexual problems due to chronic diseases. In our previous SexMEdu sub-study^25^ of 402 general practitioners, most (67%) participants considered sexual problems to be common side effects of medications prescribed for other pathologies. However, they seldom asked about possible side effects related to sexual health during follow-up consultations^25^. In a Dutch study^33^ of 204 questionnaires completed by general practitioners, 68% of participants never discussed sexual dysfunction with their chronically ill patients during the first consultation, but 54% discussed the issue during a follow-up visit. Two previous studies^34,35^ among nursing students examined this issue. Similar to our findings, a Taiwanese study^34^ of 202 nursing students showed that to address patients’ concerns about sexual health regarding illness, chronic disease and sexuality, 76% of students from a traditional four-year program and 57% of students from a two-year recurrent education undergraduate degree program reported moderate or strong learning needs. However, partly contradictory results were presented in a Turkish study^35^ of 475 nursing students, in which 66% of the participants reported knowing the association between chronic diseases and their treatments and sexuality. Nevertheless, overall, as many chronic diseases and their medications may induce sexual problems, associations with sexual problems should also be addressed in education.

Intriguingly, in our study, the most self-reported educational interests differed only slightly between the two student groups. Both groups reported a high interest in learning more about the reasons for and treatments of dyspareunia and a lack of sexual desire. In contrast, in a German study^23^ of 2928 questionnaires completed by medical students, the causes of and treatment for pain during intercourse aroused interest in only 22% of students. Regarding midwives, the previously mentioned analysis of the Swedish midwifery program^11^, showed that sexual well-being/pleasure was missing from the course syllabus. Among the medical students in our study, one of the highest educational interests was in the reasons for and treatment of erectile dysfunction, even though most considered their knowledge in the area sufficient. One reason for our finding could be the effective medications available for erectile dysfunction^36^, encouraging students to learn even more. Instead, erectile dysfunction did not arouse much interest among the midwifery students, a logical finding, as the patients of midwives are primarily women. However, even though the majority of midwives’ patients are female, at an infertility clinic, for example, a couple, not just a woman, is typically treated. Additionally, when addressing a female patient’s sexual issues, one should remember to ask about the partner’s possible sexual problems, as it can affect the woman’s sexuality too. Therefore, male sexual health issues should not be overlooked by midwives. The highest educational interest among midwifery students was the effects of infertility and its treatment on sexuality, which was also rated moderately high in the previously mentioned German study of medical students^23^. Interestingly, in the German study^23^, interest was highest in the topic of child sexual abuse (52%); in our study, however, neither group showed much interest in the topic.

In medicine, history taking is a basic step in diagnosis and treatment. In our study, the students, especially the medical students, self-reported an interest in sexual history taking. In the above-mentioned German study^23^, the percentage of students having an educational interest in sexual history taking was almost the same as ours, 32%, and the lack of knowledge of sexual history taking was also identified in the previously mentioned Austrian study^14^ of medical students, as nearly all (97%) self-reported not having being instructed in sexual history taking during medical studies. Furthermore, in a Malaysian study^16^ of 379 final-year medical students, only 27% reported having adequate skills to take a patient’s sexual history.

### Strengths and limitations

Our study has some biases but also merits. The number of students in each group in our study can be considered sufficient, even though it was smaller than in some of the previous studies in the field^23,35^. The population of Finland is small (approximately 5.5 million); thus, the annual number of students in the medical and midwifery schools is small. Accordingly, one can assume that our participant size represents the examined issue quite well. Our response rates of 41.5% and 51.8% (for medical and midwifery students, respectively) could be considered only moderate; however, compared to some previous studies with response rates from 12.3%^14^ to 70.0%^16^, our rates fall within this range. To obtain a complete perspective of the entire curriculum, we included only final-year students. With this study design, the educational level of our participants was also comparable with those of previous studies^12,16^. Because of school-wide regulations, the teachers or teaching coordinators mainly distributed the link to our questionnaire to the participants. This could have had either a negative or a positive influence on the student’s willingness to participate in our study. In addition, graduating students are typically a target of various surveys; thus, this abundance may have diminished the response rate. Not having a gender option ‘other’ in the study questionnaire might also have hindered some from participating. It is possible that students with a particular interest in sexual medicine predominantly participated in our study, biasing our results. However, if this is the case, one could have assumed more knowledge of the surveyed topics. In addition, our results may not be directly applicable to medical and midwifery students in other countries, as we included only students who studied in Finland. The original questionnaire was developed for general practitioners and medical students. Hence, the items did not cover the entire field of midwifery practice, e.g. areas such as the effect of pregnancy, labor and postpartum on sexuality. These areas would be important to investigate in the future when developing especially midwifery education. One of the merits of our study was the piloting of the study questionnaire, allowing us to revise the content. In addition, the program for our questionnaire did not allow the questionnaire to be returned without responses to items, ensuring no incomplete questionnaires; however, it could also have prevented some students from participating.

## CONCLUSIONS

Our findings emphasized a lack of education in sexual medicine in medical and midwifery education in Finland. Both medical and midwifery final-year students self-reported insufficient knowledge when assessing factors associated with sexuality and an even more profound deficiency in knowledge of evaluating and treating various sexual problems. All the educational areas regarding sexual medicine included in our study questionnaire drew at least some level of the students’ interest. Accordingly, our results pointed to the importance of allocating a wide range of topics in sexual medicine in medical and midwifery education curricula.

## Data Availability

The data supporting this research are available from the authors on reasonable request.
